# Relationship between rumen bacterial community and milk fat in dairy cows

**DOI:** 10.3389/fmicb.2023.1247348

**Published:** 2023-10-11

**Authors:** Boxue Si, Kaizhen Liu, Guoxin Huang, Meiqing Chen, Jiyong Yang, Xufang Wu, Ning Li, Wenhao Tang, Shengguo Zhao, Nan Zheng, Yangdong Zhang, Jiaqi Wang

**Affiliations:** ^1^State Key Laboratory of Animal Nutrition and Feeding, Institute of Animal Sciences, Chinese Academy of Agricultural Sciences, Beijing, China; ^2^Key Laboratory of Quality & Safety Control for Milk and Dairy Products of Ministry of Agriculture and Rural Affairs, Institute of Animal Sciences, Chinese Academy of Agricultural Sciences, Beijing, China; ^3^Henan International Joint Laboratory of Nutrition Regulation and Ecological Raising of Domestic Animal, College of Animal Science and Technology, Henan Agricultural University, Zhengzhou, China

**Keywords:** milk fat, 16S rRNA gene sequencing, rumen bacteria, fatty acid composition, key bacteria

## Abstract

**Introduction:**

Milk fat is the most variable nutrient in milk, and recent studies have shown that rumen bacteria are closely related to milk fat. However, there is limited research on the relationship between rumen bacteria and milk fatty. Fatty acids (FAs) are an important component of milk fat and are associated with various potential benefits and risks to human health.

**Methods:**

In this experiment, forty-five healthy Holstein dairy cows with alike physiological and productive conditions were selected from medium-sized dairy farms and raised under the same feeding and management conditions. The experimental period was two weeks. During the experiment, raw milk and rumen fluid were collected, and milk components were determined. In this study, 8 high milk fat percentage (HF) dairy cows and 8 low milk fat percentage (LF) dairy cows were selected for analysis.

**Results:**

Results showed that the milk fat percentage in HF group was significantly greater than that of the dairy cows in the LF group. 16S rRNA gene sequencing showed that the rumen bacterial abundance of HF dairy cows was significantly higher than that in LF dairy cows; at the genus level, the bacterial abundances of *Prevotellaceae_UCG-001, Candidatus_Saccharimonas, Prevotellaceae_UCG-003*, *Ruminococcus*_1, *Lachnospiraceae_XPB1014_group*, *Lachnospiraceae_AC2044_group*, *probable_genus_10* and *U29-B03* in HF group were significantly higher than those in the LF group. Spearman rank correlation analysis indicated that milk fat percentage was positively related to *Prevotellaceae_UCG-001, Candidatus_Saccharimonas, Prevotellaceae_UCG-003, Ruminococcus*_1, *Lachnospiraceae_XPB1014_group*, *Lachnospiraceae_AC2044_group*, *probable_genus_10* and *U29-B03*. Furthermore, *Prevotellaceae_UCG-001* was positively related to C14:0 iso, C15:0 iso, C18:0, *Ruminococcus*_1 with C18:1 t9, *Lachnospiraceae_AC2044_group* with C18:1 t9 and C18:1 t11, *U29-B03* with C15:0 iso.

**Discussion:**

To sum up, rumen bacteria in dairy cows are related to the variation of milk fat, and some rumen bacteria have potential effects on the deposition of certain fatty acids in raw milk.

## 1. Introduction

Milk fat is one of the main nutrient component in milk, and it is also a key indicator for assessing milk quality. The content of milk fat is generally 3% ~ 5% in milk, and the main component is triglyceride, which account for about 98% of milk fat. The remaining components of milk fat include diacylglycerol, monoacylglycerol, phospholipid, glycolipid, cholesterol, free fatty acids and fat-soluble vitamins (Liu et al., 2018; Amores and Virto, 2019). Fatty acids (FAs) are an important component of milk fat and are associated with various potential benefits and risks to human health. Previous studies have shown that unsaturated fatty acids reduce hypercholesterolemia and risk of cardiovascular diseases, whereas saturated fatty acids and trans fatty acids have opposite effects (Huth and Park, 2012;Valenzuela et al., 2019; Guo et al., 2023). Bovine milk contains many different fatty acids, from essential FA such as linoleic (C18:2) and α-linolenic (18,3) FA to human health promoting FA such as con-jugated linoleic acid (CLA) (C18:2) (German and Dillard, 2006; Calder, 2015). These unsaturated fatty acids have potential benefits in preventing cardiovascular disease, anticancer, anti-inflammatory, and antioxidant properties (Gómez-Cortés et al., 2018; Lawrence, 2021). Recent scientific research has shown that Branched chain fay acids and Odd-chain fatty acids are trace but important bioactive components in food. Which are gradually garnering attention from scientists for their protective effect against inflammatory and cancer (Gomez-Cortes and de la Fuente, 2020). In addition, several studies have shown that Medium chain fatty acids (MCFAs) are useful in the treatment of a variety of neurological and metabolic disorders [e.g., Alzheimer’s disease (Courchesne-loyer et al., 2017; Croteau et al., 2017), cancer (Jansen and Walach, 2016), diabetes (Geng et al., 2016), and obesity (St-Onge and Jones, 2002)] due to their immunoactivities and intestinal probiotic effects。MCFAs can be directly absorbed by intestinal epithelial cells and rapidly transferred to the liver through the portal vein, and MCFAs can cross the blood–brain barrier and preferentially undergo β-oxidation. Therefore, MCFAs are highly efficient in absorption and transportation.

Fatty acid is the main component of milk fat, and its type, composition and content have been widely concerned in the current years. The milk fatty acid was mainly synthesised by rumen microbia and exogenous uptake (Parodi, 2004). Butyric acid manufactured by microbial fermentation is absorbed and converted into β-hydroxybutyric acid by rumen epithelial cells, and acetic acid and β-hydroxybutyric acid are transported to the mammary gland to synthesize short and medium chain fatty acids. However long chain fatty acids are mainly derived from dietary lipids and body fat (Buitenhuis et al., 2019). In the context of the rumen microbial community digesting feed, it is interesting to study how much changes in milk FA can be explained by changes in the rumen microbiome.

The milk fat in milk is affected by numerous factors, such as rumen bacteria, diet, feeding management, health status, season, parity and lactation stage of dairy cows (Latham et al., 1974; Yang et al., 2013; Buitenhuis et al., 2019). In the rumen of dairy cows, bacteria can degrade plant fibers to volatile fatty acids (VFAs) by fermentation (Patil et al., 2018). Rumen bacteria have been reported to have significant influence on rumen fermentation, as well as the variation of milk yield and milk composition (Bainbridge et al., 2016; Hooman et al., 2016). The ratio of Firmicutes to Bacteroidetes was found to be positively related to milk fat yield (Jami et al., 2014; Scharen et al., 2018). Hassan et al. found that dietary regulation can increase the relative richness of Firmicutes and decrease the relative richness of Bacteroidetes, thereby increasing milk fat yield (Hassan et al., 2020). Milk fat percentage was reported the association with the abundance of *Dialister, Megasphaera, Lachnospira* and *Sharpea* in rumen (Pitta et al., 2020).

Previous studies have shown a relationship between milk fat and rumen microbiota. We speculated that the rumen bacterial composition of HF and LF groups was different, and there were differences in rumen fermentation, rumen metabolites, milk fat composition and fatty acid composition. The purpose of this study was to analyze the differences in rumen bacteria between HF and LF groups by 16S rRNA gene sequencing technology, and the relationship between differential bacteria and milk fat.

## 2. Materials and methods

### 2.1. Experimental dairy cows and feeding management

All dairy cows in this experiment were conformed to the research method, guaranteed animal welfare, and were approved by the Animal Care Committee of Institute of Animal Sciences of Chinese Academy of Agricultural Sciences (No: IAS 2019–28).

This experiment was carried out in Tianjin Mengde Dairy Farm, with more than 1,500 Holstein dairy cows in spring. The dairy farm has advanced facilities and records the milk yield of individual dairy cows every day. From more than 1,500 lactating dairy cows, 45 healthy dairy cows with similar body weight, parity (1.3 ± 0.74), day in milk (153 ± 16d) and daily milk yield (34 ± 6 kg) were selected. After collecting milk samples, MilkoScan FT120 (Foss, Hillerod) was used to determine the content of milk protein, milk fat, lactose, and total solids. The data were subjected to quality control through the mean and standard deviation (SD), and split into HF group (*n* = 8) and LF group (*n* = 8) according to the milk fat percentage. The feed intake and milk yield between the two groups were similar, which avoided the large difference in milk fat percentage caused by the huge difference in milk yield, and met the requirements of experiment.

All 45 dairy cows were raised in the same enclosure, drinking water freely and fed with TMR three times a day for two weeks. The daily feeding time was 7:00, 13:00, and 18:00, respectively, and the feeding amount and remaining feed amount of each dairy cow were recorded, and the individual feed intake was calculated. Milking took place half an hour before feeding and individual milk production was recorded by the milking system (Liu et al., 2022).

### 2.2. Raw milk and rumen fluid collection

During the experiment, the management conditions were the same. Milk samples and rumen fluid samples were taken on the last day of the experiment. Raw milk samples were collected every day according to the milking time, three times a day, 50 mL each time, stored at 4°C, and finally blended in a 4:3:3 ratio as a milk sample for subsequent milk composition determination.

The collection time of rumen fluid was before the first feeding in the morning. About 200 mL rumen fluid samples were collected before the morning feeding from each cow using oral stomach tube. The rumen fluid samples were immediately filtered using 4 layers of gauze, and then separated it into two parts. One was immediately measured for pH value, the other aliquot was poured into a 50 mL sterilized tube and stored frozen for subsequent determination of rumen bacterial composition and rumen fermentation parameters.

### 2.3. Determination of fermentation indexes

Determination of NH_3_-N and VFAs were performed after rumen fluid thawing. Analyze 16 samples for each indicator. The concentration of NH_3_-N was determined by phenol-sodium hypochlorite colorimetric method referenced the previously paper (Wang et al., 2019). The concentration of VFAs was determined by gas chromatography FID detector.

### 2.4. 16S rRNA gene sequencing

The collected rumen fluid was thawed, and then DNA extraction was performed using the cetyltrimethylammonium bromide (CTAB) reagent. The purity of extracted DNA was checked using 1% agarose gel, and the DNA concentration was determined using a NanoDrop 2000 UV–vis spectrophotometer (Thermo Scientific, Wilmington, United States). The purified DNA as template, 341F (5’-CCTACGGGNGGCWGCAG-3′) and 806R (5’-GGACTACHVGGGTWTCTAAT-3′) as primers, the V3 to V4 of bacterial 16S rRNA gene was amplified using an ABI GeneAmp 9,700 PCR thermocycler (ABI, CA, United States). The PCR amplification conditions and the specific steps of sequencing all adopt the method of Liu et al. (2022). Briefly, The PCR protocol was 94°C for 2 min, followed by 30 cycles at 98°C for 10 s and 62°C for 30 s. The PCR reaction solution consisted of 10 × KOD Buffer 5 mL, 2 mmol/L dNTP 5 mL, 25 mmol/L MgSO4 3 mL, 10 mmol/L primer 1.5 mL respectively, KOD Polymerase (TOYOBO, Japan) 1 mL, and the template DNA 100 ng. The purified products were quantified using a Quantus Fluorometer (Promega, United States), and pooled in equimolar, then pairedend sequenced (2 × 300 bp) at Illumina Hiseq 2,500 PE250 platform (Illumina, San Diego, United States) under the standard protocols by GENE DENOVO (Guangzhou, China). The raw reads of 16S rRNA gene sequencing were deposited into the NCBI Sequence Read Archive database (accession number: PRJNA722820).

All raw reads analysis and the quality control were referenced to the methods as reported by Liu et al. (2022). UPARSE (7.1 version, http://drive5.com/uparse/) was used to cluster the operational taxonomic unit (OTU) with 97% similarity cutoff, then identified, and removed the chimeric sequences. The longest read was deemed as a representative sequence of the taxonomy for each OTU. Further, those representative sequences were identified against to the Silva SSU128 database using RDP Classifier.

### 2.5. Fatty acid determination

The test was carried out by the method independently developed by our laboratory. Draw 2 mL of liquid milk sample, add 25 μL of internal standard C19:0 fatty acid methyl ester (10,000 μg/mL), add 4 mL of n-hexane/isopropanol (V/V = 3/2) mixed solution, after vortexing for 30s, centrifugation (4°C, 12,000 rpm) for 3 min. Pipette the n-hexane phase into a high temperature test tube with a lid, add n-hexane, centrifuge, take the upper n-hexane phase, repeat twice. To the n-hexane phase containing the lipid extract, 2 mL of NaOH-CH_3_OH solution (20 g/L) was added, and saponification and alkali-catalyzed methyl esterification at 50°C for 20 min in a water bath. Subsequently, Acid-catalyzed methyl esterification was carried out by adding 2 mL of acetyl chloride-methanol solution (100 mL/L) at 90°C for 120 min in a water bath. After cooling, add ultrapure water, transfer the n-hexane phase, and dilute to 10 mL. Add about 1 g of anhydrous sodium sulfate, vortex for 30 s, and let stand for 10 min. Pipette 200 μL of the n-hexane phase into the bottle and dilute the volume to 1 mL, so that the dilution factor of liquid milk to sample bottle is 25. Analysis with GC–MS.

Testing equipment: Gas chromatography tandem mass spectrometer 7890A/7000B (G-150). The conditions of use of the instrument are as follows, chromatographic column: CP-Sil 88; programmed temperature; gas: helium; constant pressure: 38 psi; inlet temperature: 250°C; quadrupole temperature: 150°C; injection volume: 1 μL.

### 2.6. Statistical analysis

Import the experimental data into Excel 2016 for preliminary statistical. Significance analysis was performed using SPSS (version 26.0). Non-parametric tests (Kruskal-Wallis) were used to compare the differences in milk composition, rumen fermentation indexes, and fatty acid composition between the two groups of cows (Nikoloudaki et al., 2019). The omicsmart^1^ microbial analysis platform was used to analyze and compare rumen bacteria, including alpha diversity analysis, PCA analysis, species composition analysis, and indicator species analysis. Correlation analysis was performed using Spearman’s rank analysis method. The relationship between rumen bacteria and milk components, NH_3_-N and VFAs, and the relationship between rumen bacteria and raw milk fatty acid composition were mainly analyzed.

## 3. Result

### 3.1. Milk composition analysis

Table 1 indicates that the day in milk and milk yield of the dairy cows in two groups were similar, and there was no statistical significance (*p* = 0.958). The CP, NDF, ADF, and DMI intakes of cows in HF group were significantly higher than that in the LF group (*P*<0.05). The milk fat percentage is the most concerned indicator. The milk fat percentage of the dairy cows in the HF group was 4.42, which was significantly greater than that in the LF group (*p* < 0.01).

**Table 1:** Feed intake, milk production, and milk composition of HF and LF dairy cows.

Item	HF	LF	SEM	*p*-value
DIM, d	153.00	152.00	4.003	0.958
Milk fat, %	4.42	2.85	0.209	0.001
Milk protein, %	3.31	3.09	0.046	0.009
Lactose, %	4.83	4.97	0.042	0.093
Non-fat milk solids, %	8.55	8.71	0.072	0.226
Total milk solid, %	12.97	11.56	0.201	0.001
Milk yield, kg/d	33.08	34.99	0.862	0.674
CP intake, kg/d	2.39	2.25	0.049	0.031
NDF intake, kg/d	13.17	12.29	0.306	0.036
ADF intake, kg/d	3.69	3.41	0.094	0.036
DMI, kg/d	21.02	19.62	0.485	0.036
Milk fat/Milk protein	1.34	0.92	0.056	0.001
TMS yield, kg/d	4.29	4.04	0.103	0.494

HF means high milk fat percentage group; LF means low milk fat percentage group; SEM means standard error of the mean; DIM means day in milk; CP means crude protein; NDF means neutral detergent fiber; ADF means acid detergent fiber; DMI means dry matter intake; and TMS means total milk solid.

### 3.2. Analysis of rumen fermentation parameters

The analysis results of the rumen fermentation indexes of dairy cows in the two groups were shown in Table 2. The pH and NH_3_-N concentration of rumen fluid between the two groups of dairy cows were similar, and the difference was not significant (*p* > 0.05). At the same time, the difference of total VFAs and individual VFAs between the two groups of dairy cows was also not significant (*p* > 0.05). However, it was worth noting that the concentration of VFAs in the rumen fluid of dairy cows in the HF group was higher than those in the LF group. In addition, the ratio of acetic acid to propionic acid and the mole percentage of each VFA between the two groups of dairy cows were not significant, and there was no statistical significance.

**Table 2:** Composition of rumen fermentation parameters in high and low milk fat percentage group.

Item	HF	LF	SEM	*p*-value
NH_3_-N, mg/dL	10.89	11.67	1.361	0.248
pH	6.90	6.73	0.074	0.318
VFA concentration, mmol/L				
Acetic acid	10.46	10.35	0.609	0.753
Propionic acid	4.19	3.94	0.342	0.916
Isobutyric acid	0.67	0.61	0.062	0.713
Butyric acid	2.87	2.70	0.343	0.916
Isovaleric acid	0.46	0.40	0.079	0.958
Valerate acid	0.37	0.36	0.027	0.833
Acetate to propionate ratio	2.58	2.69	0.092	0.753
Total VFA	19.02	18.38	1.409	0.834
Molar proportion, %				
Acetic acid	0.56	0.57	0.011	0.712
Propionic acid	0.22	0.21	0.004	0.957
Isobutyric acid	0.03	0.03	0.001	0.811
Butyric acid	0.14	0.14	0.007	0.749
Isovaleric acid	0.02	0.02	0.002	0.907
Valerate acids	0.02	0.02	0.001	0.317

HF means high milk fat percentage group; LF means low milk fat percentage group; SEM means standard error of the mean; NH_3_-N means ammonia nitrogen; and VFA means volatile fatty acid.

### 3.3. Analysis of rumen bacterial richness

**Figure 1:**
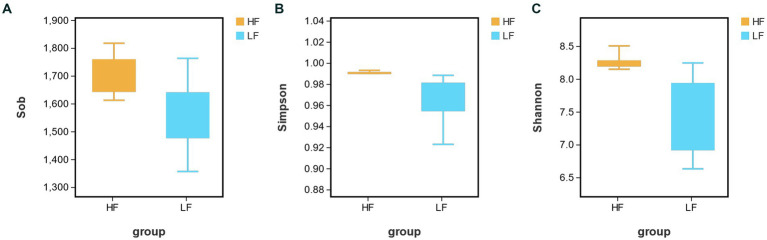
Analysis of rumen bacterial richness index [**(A)** Sobs; **(B)** Simpson; and **(C)** Shannon] in HF group and LF group. HF means high milk fat percentage; LF means low milk fat percentage.

16S rRNA gene sequencing analysis of rumen fluid samples from 16 cows obtained a total of 1,624,665 sequences, with an average of 10,1,541 ± 4,613 sequences per sample. After the sequencing results were processed for quality control, the bacterial richness index was obtained by using the alpha diversity analysis of rumen bacteria. The results in Figure 1 showed that the bacterial abundance indices Sobs, Simpson and Shannon in the HF group were significantly higher than LF group (*p* < 0.05, *p* < 0.05, *p* < 0.01). It indicated that there is a higher abundance of bacteria in the HF rumen.

**Figure 2:**
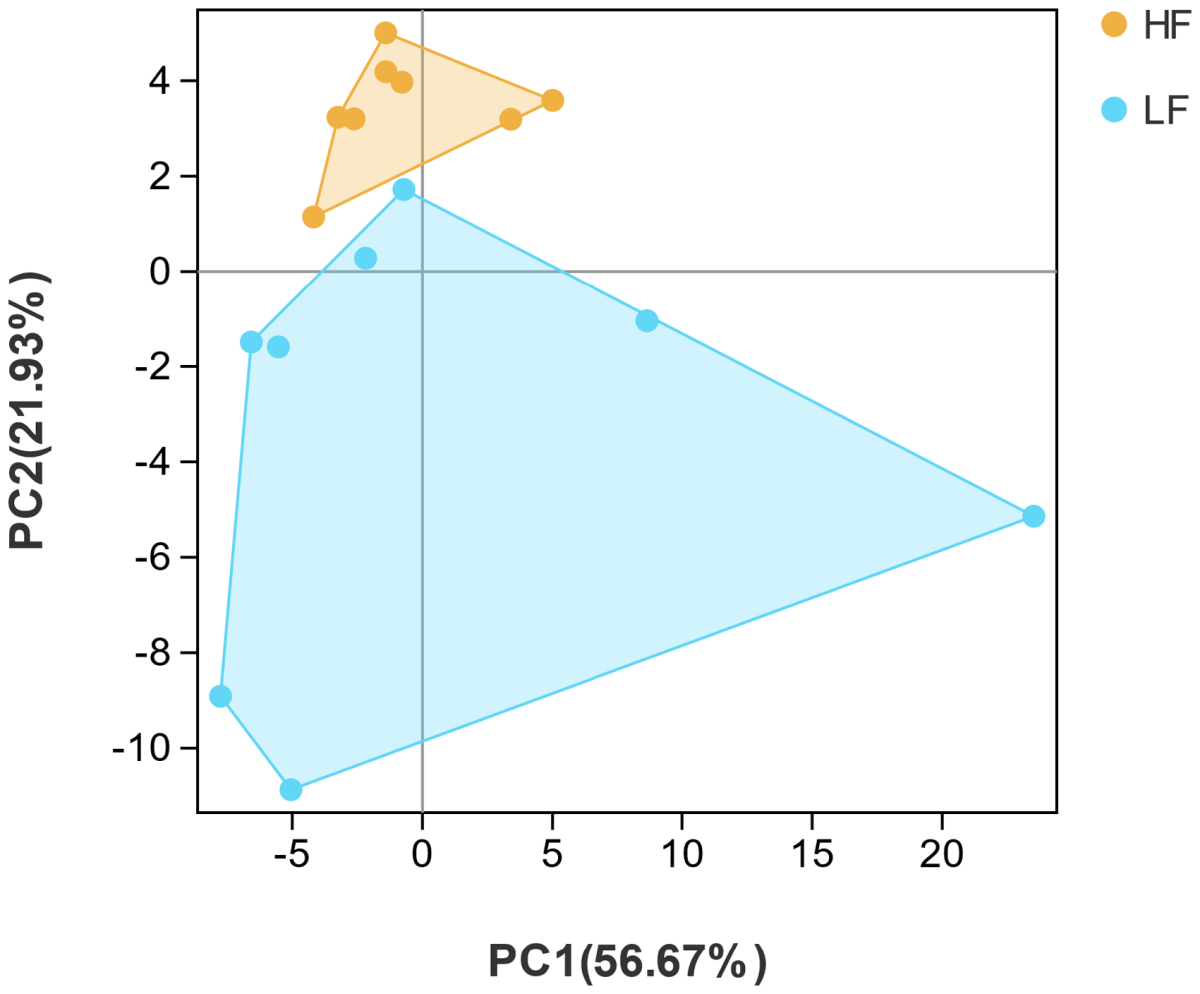
Principal component analysis (PCA). PCA analysis of rumen bacteria in HF group and LF group. HF means high milk fat percentage; LF means low milk fat percentage.

By listing species abundance information based on OTUs, a PCA plot was carried out to show the similarity between and within the two groups of dairy cows. The PCA diagram indicated that the variation degrees of the PC1 and PC2 were 56.67 and 21.93%, respectively. Moreover, the rumen bacterial composition of HF group and LF group dairy cows was clearly distinguished, indicating the rumen bacterial structure was significantly different between HF group and LF group (Figure 2). Samples with high community structure similarity in PCA plot tended to cluster together, whereas the greater the community difference, the farther the distance. It could be seen that the structural similarity of the rumen flora of dairy cows in the HF group was significantly greater than that in the LF group.

### 3.4. Analysis of the rumen flora structure

**Figure 3:**
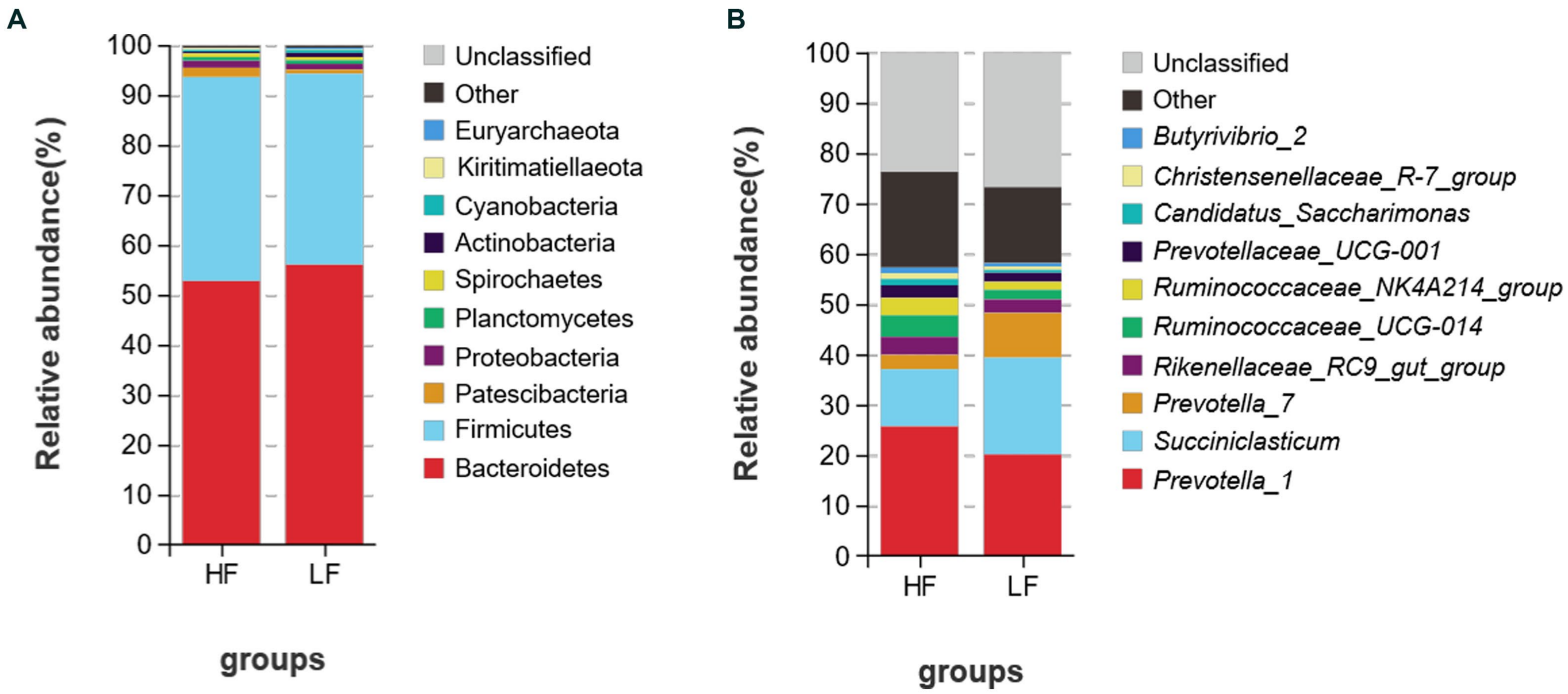
Relative abundance of rumen bacteria at the phylum **(A)** and genus **(B)** levels of dairy cows in HF group and LF group. HF means high milk fat percentage; LF means low milk fat percentage.

A total of 25 bacterial phyla were discovered at the phylum level after classification analysis. Among them, the relative abundance of Bacteroidetes and Firmicutes accounted for more than 93.0% (Figure 3A). The ratio of Firmicutes to Bacteroidetes was 0.873 in the HF group and 0.724 in the LF group. As shown in Figure 3B, a total of 225 bacteria were detected at the genus level, and the relative abundance of *Prevotella_1* was the highest, and both groups account for more than 20% of the total bacteria, followed by *Succiniclasticum,* and *Prevotella-7*. LF had 1.69 times more *Succiniclasticum* than HF (HF 11.39%, LF 19.22), *Prevotella_7* was 3.14 times higher than HF (HF 2.83%, LF 8.89%).

**Figure 4:**
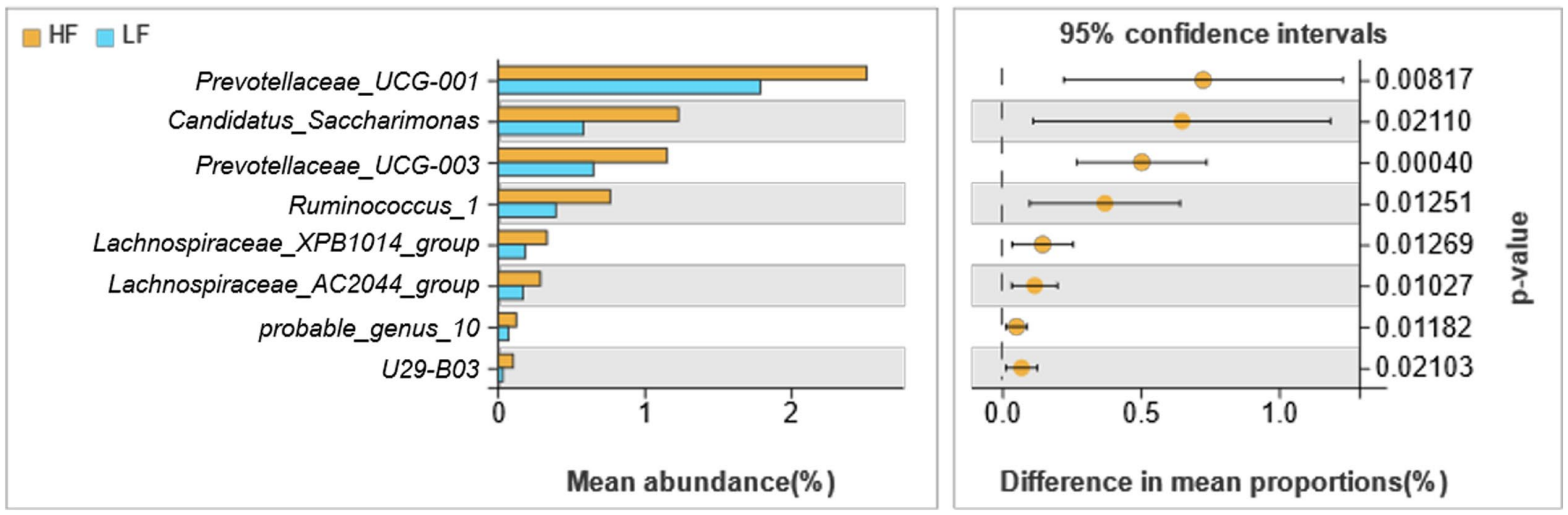
Comparison of rumen bacteria in dairy cows in HF group and LF group. HF means high milk fat percentage; LF means low milk fat percentage.

There were 8 different genera of bacteria between the two groups, which basically belong to Bacteroidetes and Firmicutes (Figure 4). The relative abundance of *Prevotellaceae_UCG-001*, *Candidatus_Saccharimonas, Prevotellaceae_UCG-003*, *Ruminococcus*_1*, Lachnospiraceae_XPB1014_group*, *Lachnospiraceae_AC2044_group*, *probable_genus_10* and *U29-B03* of rumen fluid in HF dairy cows were significantly greater than those in LF dairy cows (*p* < 0.05).

### 3.5. Correlation analysis of milk composition and fermentation index

**Figure 5:**
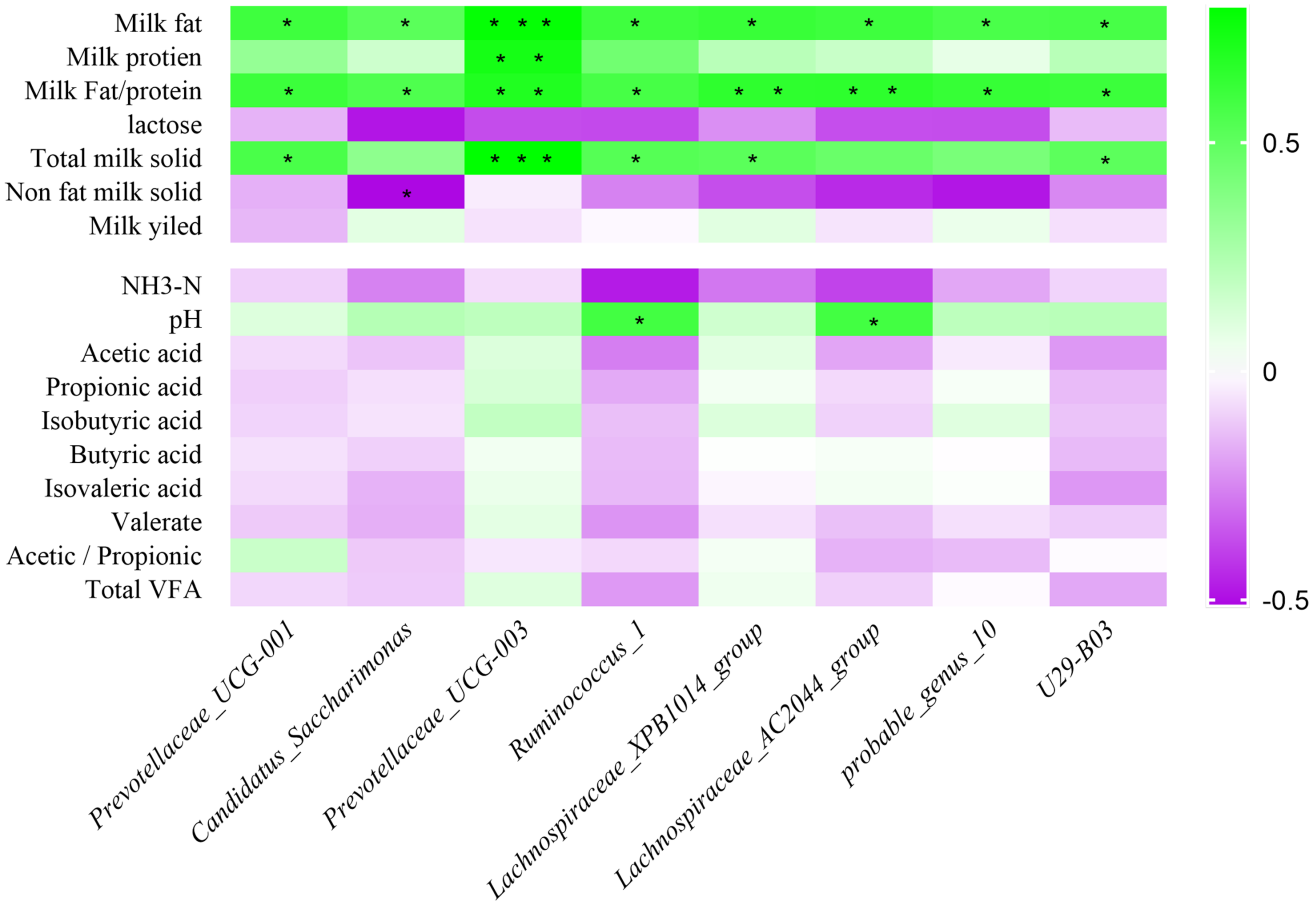
Correlation analysis of rumen bacteria with milk components and fermentation parameters. Green represents positive correlation and purple represents negative correlation. The color depth represents the correlation coefficient value, and the darker the color, the larger the value. NH_3_-N means ammonia nitrogen; VFA means volatile fatty acid; *represents a significant difference (0.01 < *p* < 0.05). ** represents (0.001 < *p* < 0.01), *** represents (*p* < 0.001).

Correlations between milk composition, rumen fermentation indexes, and relative abundance of rumen bacteria were analyzed in HF and LF dairy cows using Spearman rank correlation analysis. The result is shown in Figure 5, *Prevotellaceae_UCG-001, Candidatus_Saccharimonas, Prevotellaceae_UCG-003*, *Ruminococcus*_1*, Lachnospiraceae_XPB1014_group*, *Lachnospiraceae_AC2044_group*, *probable_genus_10* and *U29-B03* bacterial relative abundance were significantly positive correlated with milk fat percentage, and all of them were negatively correlated with non-dairy solids and lactose content. Among the rumen fermentation indexes, the relative abundance of 8 rumen differential bacteria were all positively related to pH, but inversely correlated with NH_3_-N. The relative abundance of *Prevotellaceae_UCG-003* was positively related to VFAs.

### 3.6. Milk FA composition

**Figure 6:**
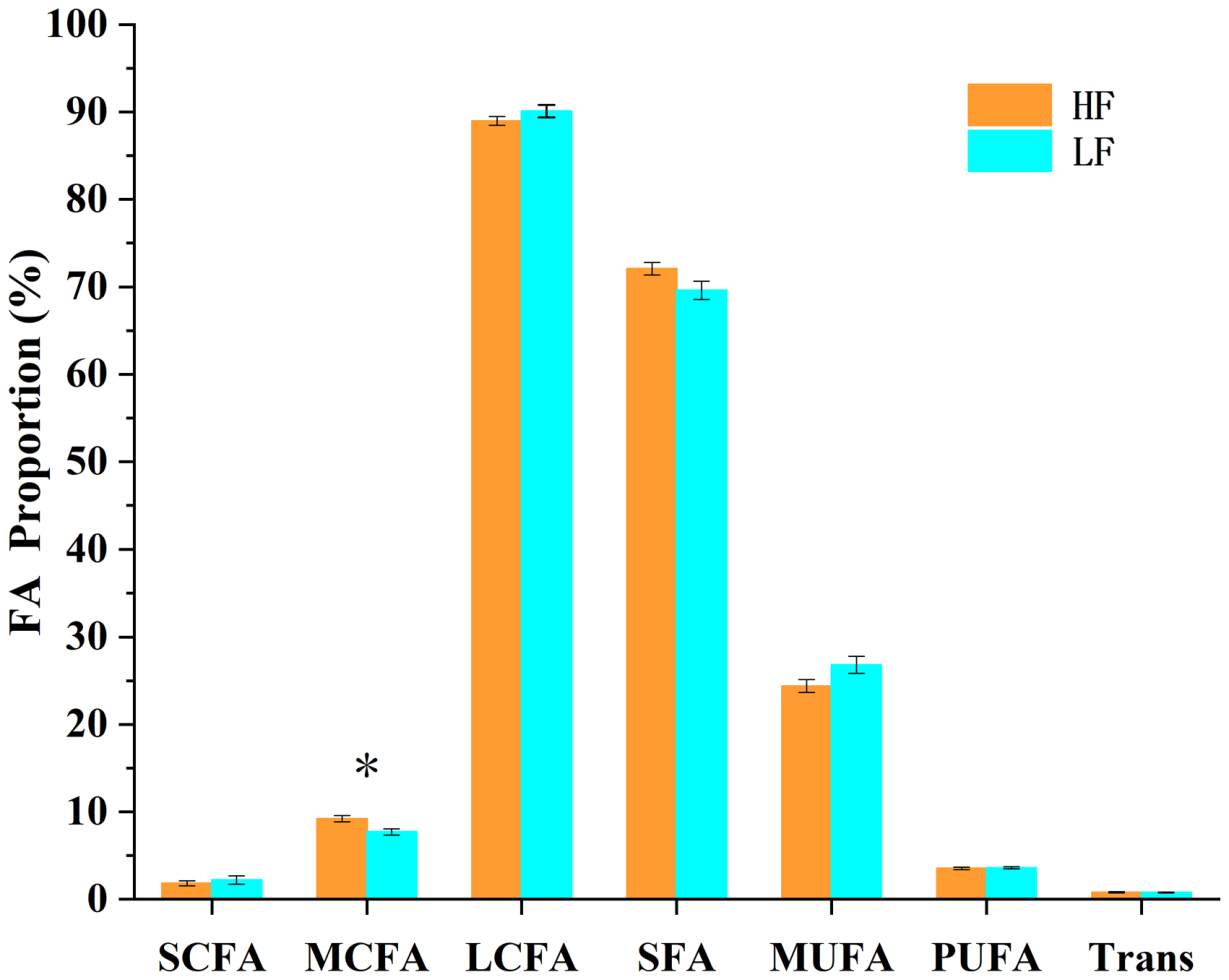
Different fatty acid groups in the high and low milk fat rate groups. SCFA: short chain fatty acids (<6 carbons), MCFA: medium chain fatty acids (6–12 carbons), LCFA: long chain fatty acids (>12 carbons), SFA: saturated fatty acids, MUFA: monounsaturated fatty acids, PUFA: polyunsaturated fatty acids, Trans: total trans fatty acids. The error line is standard error of the mean. * represents a significant difference (*p* < 0.05).

A total of 22 fatty acids were significantly different (*p* < 0.05) between the two groups of cows (Table 3; Figure 6). C7:0, C8:0, C9:0, C10:0, C11:0, C12:0, C13:0, and MCFA were significantly higher (*p* < 0.05) in cows in the high milk fat group than in cows in the low milk fat group. In contrast, cows in the high milk fat group had C16:1 c7, C17:1 t10, C18:1 c9, C18:3 c6, c9, c12, C20:1 c11, C20:2 c11, c14, C22:0, C22:1 c13, C20:3 c11, c14, c17, C20:4 c5, c8, c11, c14, C20:5 c5, c8, c11, c14, c17, C24:0, C22:4 c7, c10, c13, c16, and C22:5 c7, c10, c13, c16, c19 were significantly lower (*p* < 0.05) than cows in the low milk fat group. SCFA, LCFA, SFA, MUFA, PUFA, and Trans contents were not significantly different (*p* > 0.05) between the two groups.

**Table 3:** Differential milk fatty acid composition in high and low milk fat percentage groups.

Fatty acid	HF	LF	SEM	*p*-value
C7:0	0.01	0.00	0.002	0.027
C8:0	1.16	1.00	0.040	0.046
C9:0	0.04	0.02	0.005	0.012
C10:0	2.59	2.10	0.103	0.016
C11:0	0.03	0.00	0.007	0.004
C12:0	2.87	2.32	0.116	0.024
C13:0	0.06	0.03	0.007	0.005
C16:1 c7	0.14	0.15	0.005	0.079
C17:1 t10	0.01	0.02	0.001	0.009
C18:1 c9	20.04	22.40	0.616	0.036
C18:3 c6, c9, c12(GLA)	0.04	0.05	0.002	0.005
C20:1 c11	0.05	0.06	0.002	0.041
C20:2 c11, c14	0.03	0.04	0.002	0.004
C22:0	0.02	0.03	0.001	0.014
C22:1 c13	0.05	0.07	0.003	0.009
C20:3 c11, c14, c17(ω-3)	0.03	0.04	0.002	0.009
C20:4 c5, c8, c11, c14(AA)	0.06	0.07	0.002	0.009
C23:0	0.03	0.04	0.002	0.021
C20:5 c5, c8, c11, c14, c17(EPA/ω-3)	0.04	0.05	0.002	0.014
C24:0	0.02	0.03	0.001	0.014
C22:4 c7, c10, c13, c16	0.05	0.06	0.003	0.008
C22:5 c7, c10, c13, c16, c19(DPA/ω-3)	0.05	0.07	0.003	0.006

HF means high milk fat percentage group; LF means low milk fat percentage group; and SEM means standard error of the mean.

### 3.7. Correlation analysis between rumen bacteria and fatty acids

**Figure 7:**
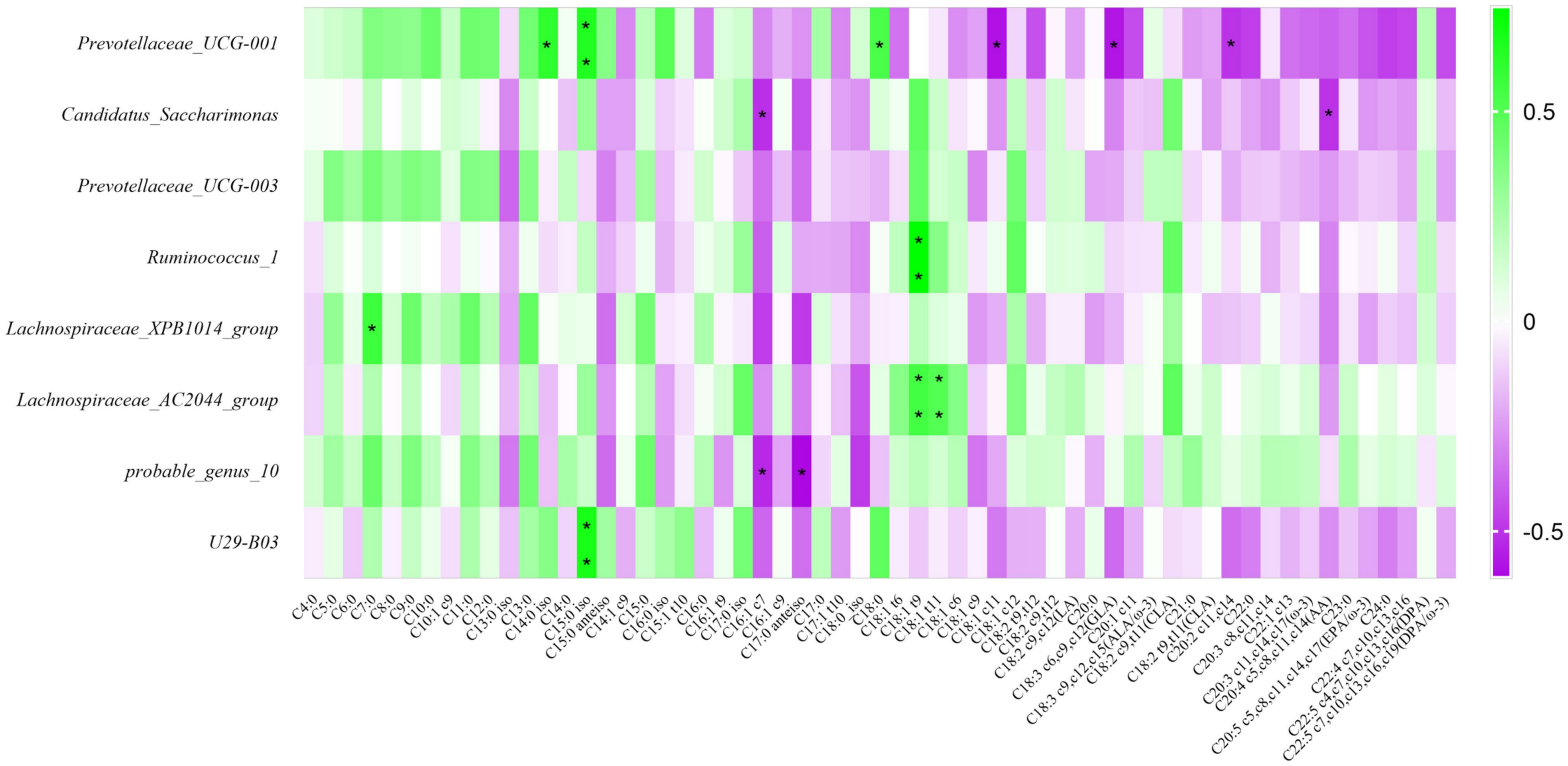
Correlation analysis of rumen bacteria with milk components and fermentation parameters. Green represents positive correlation and purple represents negative correlation. The color depth represents the correlation coefficient value, and the darker the color, the larger the value. *represents a significant difference (0.01 < *p* < 0.05). **represents very significant difference (*p* < 0.01).

Detecting fatty acids in raw milk by GC–MS, we determined a total of 59 fatty acids, and carried out correlation research with fatty acids. The result was shown in Figure 7, the relative abundance of *Prevotellaceae_UCG-001* was positively related to C14:0 iso, C15:0 iso, and C18:0 (*p* < 0.05); the relative abundance of *Ruminococcus*_1 was positively related to C18:1 t9 (*p* < 0.05); the relative abundance of *Lachnospiraceae_XPB1014_group* was positively related to C7:0 (*p* < 0.05); The relative abundance of *Lachnospiraceae_AC2044_group* was positively related to C18:1 t9 and C18:1 t11 (*p* < 0.05); The bacterial abundance of *U29-B03* was positively correlated with the C15:0 iso (*p* < 0.05). Summarily, these 8 differential bacteria were positively correlated with short and medium chain fatty acids. However, it was inversely correlated with long chain fatty acids.

## 4. Discussion

In this study, the rumen bacterial composition, milk composition and rumen fermentation indexes of dairy cows in HF and LF groups were investigated. Few previous studies in this area have been reported. A recent study showed that dairy cows in the high milk solids group had a significantly higher percentage of milk fat than cows in the low milk solids group (Liu et al., 2022). We also acquired the same research results and similar findings were also found by Nichols et al. (2018). Milk fat is affected by many factors, among which the interaction of diet structure and rumen fermentation was one of the main reasons (Buitenhuis et al., 2019; Wang et al., 2019; Xue et al., 2020). In our results, the pH value and NH_3_-N concentration indicated that the rumen maintained a stable acid–base environment, and the molar percentage of VFAs composition indicated that the rumen fermentation model of the dairy cows did not change. And, no differences were found in rumen fermentation indicators between HF and LF group dairy cows. This was difficult to explain the difference in milk fat between the two groups of cows. But research has found that the NDF and ADF feed intake of HF was significantly higher than that in LF dairy cows (13.4% and 14.4%, respectively). Plant fibers are decomposed by rumen bacteria into VFAs and other precursors for synthetic milk fat (Patil et al., 2018). This may explain why the milk fat percentage of dairy cows in the HF was greater than that in the LF.

Recently, it has been reported that rumen bacteria affect milk composition in dairy cows (Zeng et al., 2019; Zou et al., 2019). In this study, 16S rRNA gene sequencing technology was used to investigate rumen bacteria in dairy cows. It was found that the rumen bacterial richness of dairy cows in the HF was obviously higher than that of the dairy cows in the LF. Xue et al. (2018) found that the lower the richness of the rumen microflora, the lower the milk fat percentage, which is in line with the current study. It was reported that rumen microbial abundance was negatively correlated with feed efficiency and milk fat percentage (Shabat et al., 2016; Li and Le, 2017; Xue et al., 2020). This indicated that the concentration of total VFAs and individual VFAs in LF rumen should be higher than HF, however the results were opposite. We speculated that this may be related to the high number and structural similarity of rumen bacteria in HF dairy cows, resulting in higher rumen fermentation efficiency and fermentation products. In addition, the concentration of rumen VFAs was the common result of microbial fermentation and rumen absorption, and individual absorption differences may also affected. Future studies on volatile fatty acids absorption and its relationship to rumen bacteria should be investigated, which has implications for improving milk fat.

It was found that there were differences of the rumen bacteria between the two groups through the analysis of rumen bacterial composition (Jami et al., 2014). Jami et al., found that the ratio of Firmicutes and Bacteroidetes had extremely significant positive correlation with milk fat, which exhibited the same result as this study. The mean value of Firmicutes/Bacteroidetes in HF group was 0.873, which was greater than that in LF group, 0.724, and there are significant differences. *Prevotella* was reported to be the most abundant genus in the rumen of dairy cows at the genus level (Lima et al., 2014; Xue et al., 2019; Liu et al., 2022), and the same result was found in this study. The same was found in buffalo, where *Prevotella* was also the predominant genus (Lin et al., 2015; Iqbal et al., 2018). In addition, *Succiniclasticum* was the second most abundant. *Succiniclasticum* ferments carbohydrates to produce succinate, which was the precursor of propionic acid (Wallace et al., 2015). Based on our results, the propionate concentration of HF dairy cows was higher than that of LF, thus we speculated that the abundance of *Succiniclasticum* in HF dairy cows was greater than that of LF. However, the rumen bacterial results showed that Vibrio succinates in low-LF was 1.69 times higher than HF, which was inconsistent with our speculation. Xue et al., also found the same result in their study (Xue et al., 2019). This difference may be due to imprecise taxonomic assessments only at the genus level.

In-depth research on rumen microorganisms not only improve rumen digestion and feed utilization efficiency, but also provide a theoretical basis for improving milk fat and milk yield (Firkins and Yu, 2015). We focused on the analysis on differences at the genus level, specific bacteria that may be associated with milk fat. In previous reports, *Lachnospiraceae* and *Ruminococcaceae* were important bacteria for fermentative production of volatile fatty acids (Biddle et al., 2013). We found that *Lachnospiraceae_XPB1014_group*, *Lachnospiraceae_AC2044_group*, *probable_genus_10* were positively related to milk fat percentage. In addition, *Lachnospiraceae_XPB1014_group*, *probable_genus_10*, *Prevotellaceae_UCG-003* were positively related to isobutyric acid and propionic acid, Liu et al., also had the same findings (Liu et al., 2022). Zou et al.’s study on buffalo found that *Ruminococcus* was negatively related to the content of butyric acid, and we also had the same finding (Zou et al., 2019). Jami et al., and Jiang et al., found that the relative abundance of *Prevotella* was inversely related to milk fat (Jami et al., 2014; Jiang et al., 2017). However, Xue et al., found that *Prevotella* could produce higher VFAs (Xue et al., 2019). Our results found that *Prevotellaceae_UCG-003* was positively correlated with VFAs, while *Prevotellaceae_UCG-001* was negatively correlated, which may be due to the more detailed classification. Jewell et al. had found that there were functional differences between bacteria of the same genus (Kelsea et al., 2015). In this study, it was also found that *Prevotellaceae_UCG-001, Candidatus_Saccharimonas, Prevotellaceae_UCG-003, Ruminococcus_1*, *U29-B03* were all positively correlated with milk fat. Acetate is reported to be the main substrate for milk fat synthesis in the rumen of dairy cows (Xue et al., 2020). These bacteria may be positively correlation with acetate production (Xue et al., 2020; Liu et al., 2022).

Milk quality is closely related to the composition and content of fatty acids. High quality milk should be rich in unsaturated fatty acids, short chain fatty acids and medium chain fatty acids, with a corresponding reduction in saturated fatty acids and trans fatty acids (Chen et al., 2023). Saturated fatty acids can reduce people’s risk of cardiovascular disease, but unsaturated and trans fatty acids can increase the risk. Short chain and medium chain fatty acids both have anti-inflammatory, tumor growth inhibition, and obesity alleviation functions (Gómez-Cortés et al., 2018; Prado et al., 2019). In this study, it was found that the medium chain fatty acid content of cows in the HF group was significantly higher than that of the LF group, which is favorable for the production of high quality milk. Rumen bacteria break down plant fibers into volatile fatty acids (especially acetic acid and butyric acid) to synthesize medium chain fatty acids (Prado et al., 2019). The high abundance of rumen bacteria in the HF group provided the basis for the production of large amounts of medium chain fatty acids. In addition, although there was no significant difference between saturated and unsaturated fatty acids, cows in the HF group had higher levels of saturated fatty acids than those in the LF group and lower levels of unsaturated fatty acids than those in the LF group. This may be due to the conversion of unsaturated fatty acids to saturated fatty acids by rumen bacterial hydrogenation (Koch and Elascano, 2018).

To reveal the effect of rumen bacteria on milk fat, we further analyzed the association of rumen bacteria with the fatty acid composition of raw milk. Our results showed that *Prevotellaceae_UCG-001*, *Candidatus_Saccharimonas, Prevotellaceae_UCG-003*, *Ruminococcus_1*, *Lachnospiraceae_XPB1014_group*, *Lachnospiraceae_AC2044_group*, *probable_genus_10* and *U29-B03* were favorable for the deposition of short and medium chain fatty acids in raw milk, but not for long chain fatty acids. The reason may be due to the potential biohydrogenation of these rumen bacteria. It had been reported that *Lachnospiraceae* and *Prevotellaceae* were the major biohydrogenation bacteria in the rumen of dairy cows. It was noteworthy that, *Prevotellaceae_UCG-001* promoted deposition of C14:0 iso, C15:0 iso, C18:0; *Ruminococcus_1* could promote deposition of C18:1 t9; *Lachnospiraceae_AC2044_group* could promote the deposition of C18:1 t9 and C18:1 t11; *U29-B03* could promote the deposition of C15:0 iso. Bart et al. studied the relationship between rumen microbes and fatty acid composition of raw milk. The results showed that bacteria such as *Prevotella*, *Lachnospiraceae*, *Ruminococcus* could affect the synthesis of C18 unsaturated fatty acids and odd-chain fatty acids (Buitenhuis et al., 2019), and we also had the same result. Branched-chain fatty acids have the same active function of inhibiting inflammation and suppressing tumor growth (Mika et al., 2016). It was of great significance to human health. *Prevotellaceae_UCG-001* and *U29-B03* can be considered as rumen bacteria that improve milk quality. It was of practical significance to study the relationship between rumen bacterial composition and fatty acid synthesis, especially beneficial fatty acid synthesis, for improving milk quality and protecting human health.

## 5. Conclusion

There were significant differences of the rumen microbial flora diversity and the abundance in dairy cows between HF and LF. The richness of rumen bacteria in the HF was significantly greater than that in the LF, and the HF had a similar microbial community structure and little difference in colonies. *Prevotellaceae_UCG-001*, *Candidatus_Saccharimonas*, *Prevotellaceae_UCG-003*, *Ruminococcus_1*, *Lachnospiraceae_XPB1014_group*, *Lachnospiraceae_AC2044_group*, *probable_genus_10* and *U29-B03* bacterial were significantly positive correlated with milk fat percentage. And, *Prevotellaceae_UCG-003* could increase the production of VFAs. In addition, we also found that *Prevotellaceae_UCG-001* had significant deposition effects on C14:0 iso, C15:0 iso, C18:0, and *Lachnospiraceae_AC2044_group* on long-chain fatty acids C18:1 t9, C18:1 t11.

## Data availability statement

The datasets presented in this study can be found in online repositories. The names of the repository/repositories and accession number(s) can be found at: https://www.ncbi.nlm.nih.gov/, PRJNA722820.

## Ethics statement

The animal studies were approved by Animal Care Committee of Institute of Animal Sciences of CAAS (Beijing, China). The studies were conducted in accordance with the local legislation and institutional requirements. Written informed consent was obtained from the owners for the participation of their animals in this study.

## Author contributions

BS: investigation, data curation, and writing – original draft. KL and GH: methodology and validation. MC and XW: formal analysis. JY, NL, and WT: investigation. YZ: conceptualization and writing – review. SZ: conceptualization and project administration. NZ: investigation and writing – review and editing. JW: supervision, conceptualization, and writing – review and editing. All authors contributed to the article and approved the submitted version.

## Footnotes

^1^ http://www.omicsmart.com
